# Clinical analysis of posterior segment injury in left-behind children in China

**DOI:** 10.1186/s12886-020-1317-5

**Published:** 2020-01-31

**Authors:** Zhongcui Sun, Xiaonan Zhuang, Gezhi Xu, Rui Jiang

**Affiliations:** Eye and ENT Hospital of Fudan University, Fenyang Road, No 83, Shanghai, China

**Keywords:** Posterior segment injury, Left-behind children (LBC), Guarded children, Transconjunctival sutureless vitrectomy

## Abstract

**Background:**

We aimed to evaluate the severity and prognosis of posterior segment injury between left-behind children (LBC) and guarded children (NLBC).

**Methods:**

A retrospective, controlled analysis of a case series was performed. Patients diagnosed with posterior segment injury in Department of vitreous and retinal, the Affiliated Ophthalmology and Otolaryngology Hospital of Fudan University were included in this study. Patients were divided into two groups, including LBC group (*n* = 48) and NLBC group (*n* = 44). All the children underwent 25G transconjunctival sutureless pars plana vitrectomy.

**Results:**

Compared with NLBC, LBC had delayed treatment, worse baseline vision and visual prognosis, lower OTS rating, more times of vitrectomies, more complicated surgical procedures, and higher rate of lens removal and silicone oil tamponade.

**Conclusions:**

Due to lack of care and delayed treatment, posterior segment ocular trauma in the LBC was more severe, more common complicated with infectious endophthalmitis, and had worse visual prognosis. It was urgent to enforce the guardianship in LBC.

## Background

Ocular trauma is the main cause of damage to children’s vision [1, 2], which not only severely affects children’s visual development and physical and mental health, but also causes great loss to family and society. The incidence rate of paediatric ocular trauma in developing countries accounts for 1 in 1000 [3, 4]. The blindness rate of paediatric ocular trauma in China is as high as 22.4 to 35.9% [5, 6]. With the acceleration of China’s urbanization and the increasing of population migration, more than 40 million left-behind children (LBC) in rural areas are lack of effective supervision. Therefore, preventing the blindness of LBC still has a long way to go.

Paediatric ocular trauma is often involved in the posterior segment of the eye, accompanied by multiple tissue damage, irreversible damage to visual function, which brings great difficulty to treatment. Twenty-three gauge minimally invasive pars plana vitrectomy (25G-PPV) is an effective method for the treatment of paediatric ocular trauma of the posterior segment. 25G-PPV shows benefitss in small wound, short operation time, light postoperative reaction, rapid anatomic structure and rapid visual function recovery [7].

The purpose of this study is to compare the severity and prognosis of ocular posterior segment trauma between LBC and guarded children (none LBC, NLBC), and to clarify the characteristics of ocular posterior segment trauma and surgical prognosis of left-behind children.

## Methods

### Study design

A retrospective review of children (aged< 16) with posterior segment injury presenting to the Department of vitreous and retinal between Jan 2012 and Jan 2015 was performed. This study was approved by the Institutional Review Board. Cases that underwent 25G minimally invasive vitrectomy were identified and patients with follow-up of 18 months or more were included in this study.

Data included the patient’s baseline character (gender, age), eye condition (visual acuity, trauma type, retinal detachment and intraocular infection etc.) and surgical methods (whether lens removal, intraocular lens (IOL) implantation, intraocular tamponade, or enucleation etc.). we divided visual acuities into 5 grades: No light perception (NLP), light perception/hand move (LP/HM), count finger to 0.1 (CF to 20/200), 20/200 to 20/50, and ≥ 20/40.

### Definition of LBC

According to previous studies [8, 9], LBC were defined as children who were under 18 years old and were left at home with both or one of their parents migrate to urban areas for at least 6 months. Children with one of their patients leaving away for less than 6 months were defined NLBC.

Severity of ocular trauma of all patients were evaluated using the ocular trauma score (OTS) [10], and the OTS score was calculated according to the factors affecting vision. OTS-1 (0–44 points); OTS-2 (45–65 points); OTS-3 (66–80); OTS-4 (81–91); OTS-5 (92–100).

### Surgery procedure

25G-PPVs were performed in all children. In cases with traumatic cataract or rupture of the capsule, lens was removed using phacoemulsification. At the end of surgery, long effect gas C3F8 (perfluorocarbon) or silicone oil was used for intraocular tamponade, depending on the damage of the retina. For cases with endophthalmitis, ceftazidime (2.25 mg/0.1 ml) and norvancomycin (1.0 mg/0.1 ml) were given. Surgical indications included: 1) I, II area trauma accompanied with vitreous, and retina traction; 2) III area trauma accompanied with retinal incarcerated or retinal traction; 3) traumatic retinal detachment, including serrated and tractional /rhegmatogenous retinal detachment, and complex retinal detachment with retinal incarceration; 4) vitreous hemorrhage after trauma obscures the macula, which is not absorbed after a month [11].

### Statistical analysis

Data were analyzed using Statistical Product and Service Solutions (SPSS) software version 15, (IBM Corp., Armonk, NY, USA). The independent t test and Fisher’s exact test were performed to evaluate the data. A *P* value< 0.05 was considered statistically significant.

## Results

### Baseline characteristics

A total of 92 cases (92 eyes) at the Affiliated Ophthalmology and Otolaryngology Hospital of Fudan University underwent vitreous surgery treatment, including left-behind children (*n* = 48) and guarded children (*n* = 44), 50 cases were male, 42 cases were female, as show in Table 1. Age range 2–16 years old, mean patient age was 8.5 ± 4.6 years. Twenty-seven (61.4%) patients had open globe injuries, four (9.1%) patients had intraocular foreign body, six (13.6%) patients had closed globe injury, all from the guarded group. The follow-up time ranged from 18 to 32 months, and no one was lost. LBC had a higher incidence of infective endophthalmitis than NLBC. LBC are more likely to develop Relative Afferent Pupillary Defect (RAPD) than NLBC, and OTS rating is worse than NLBC. Totally two cases in LBC group and one case in NLBC group was excluded. One case in LBC group had been operated before using 20G PPV (sterilized air tamponade) in another hospital. The other case in LBC group could not afford the travelling expenses and was advised to be followed-up in the hospital in the vicinity of his home. The excluded case in NLBC group was a 12 years-old boy and had intraocular foreign body (IOFB) without lens or retinal damage. The child received simple 25G-PPV and IOFB removal, and his visual acuity recovered to 20/40 one week after surgery. He was followed up for a total of 7 months until he was sent abroad for junior high school. These three children were followed up by telephone and never come back to our department, therefore they were excluded from our study.

**Table 1:** Demographics of left-behind children and guarded children

Variables	LBC group (*n* = 48)	NLBC group (*n* = 44)	Total	*P*
Age, mean ± SD	8.7 ± 4.8	8.3 ± 2.9	8.5 ± 4.6	0.323
Gender, n				0.971
Male	26	24	50	
Female	22	20	42	
OTS,n(%)				
1	18 (37.5%)	12 (27.3%)	30	< 0.05
2	24 (50.0%)	20 (45.5%)	44	
3	6 (12.5%)	9 (20.5%)	15	
4	0	3 (6.8%)	3	
5	0	0	0	
Complications, n(%)				
Eyeball Rupture	5 (10.4%)	10 (22.7%)	15	0.110
Infectious endophthalmitis	18 (37.5%)	1 (7.1%)	19	0.000
Perforating injury of the eyeball	0	0	0	
Retinal detachment	27 (56.3%)	25 (56.8%)	52	0.414
RAPD	42 (87.5%)	21 (47.7%)	63	0.000

Fisher’s exact test was conducted to compare the indexes

### Visual outcomes

**Fig. 1:**
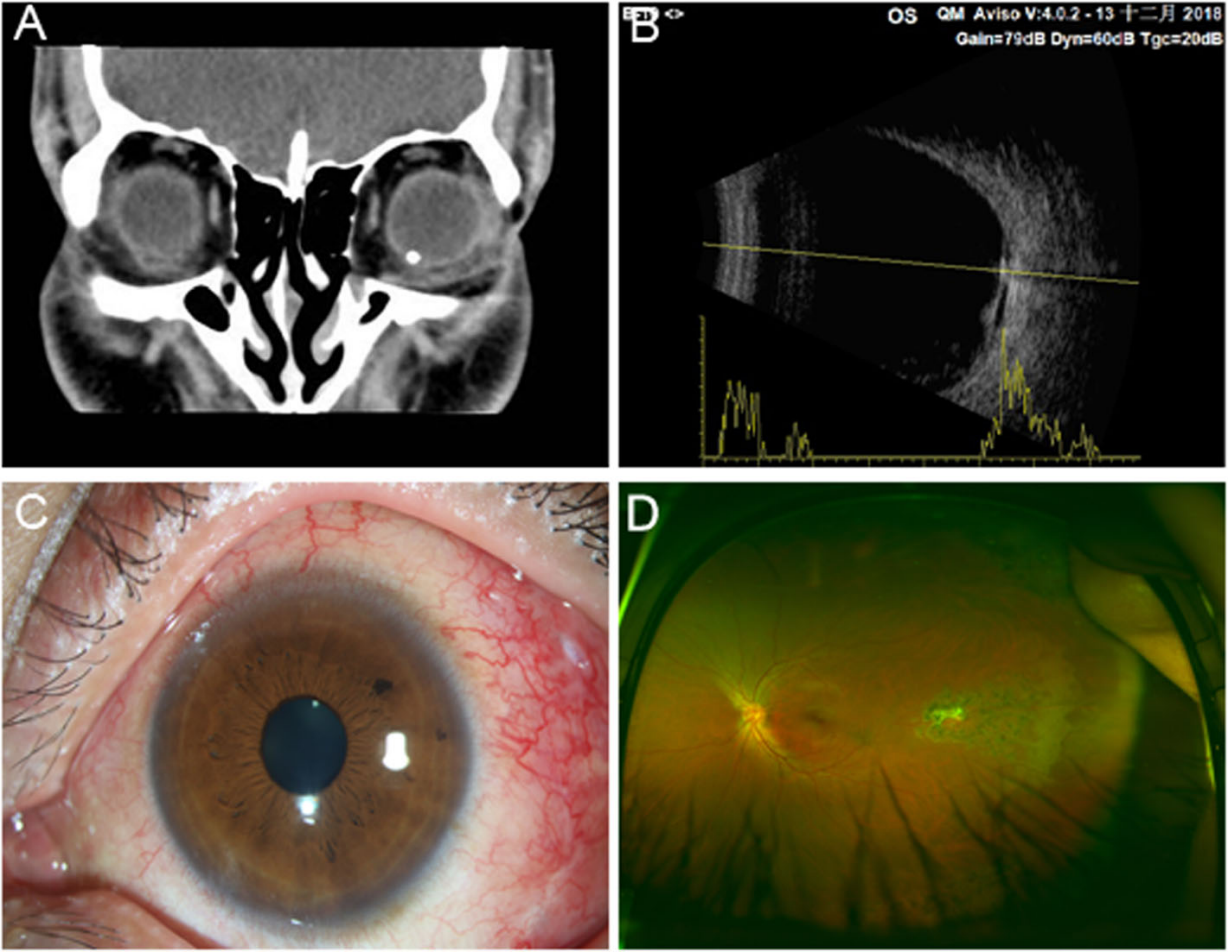
A patient with positive foreign body in the bulb of the left eye. (**a**) Preoperative coronal CT scan of the left eye. (**b**) Preoperative transverse CT scan of left eye. (**c**) Postoperative left anterior segment showed conjunctival congestion in the left eye, clear cornea, sensitive pupil response to light, transparent lens. (**d**) Left eye fundus photography after surgery showed vitreous fluid filling in left eye, retinal flattening, old laser spot in temporal macular area, White scar in the central laser spot

The comparison of the initial and final visual acuity between LBC and NLBC is shown in Table 2. In terms of composition ratio, the proportion of the initial and final eyesight of LBC above the index is significantly smaller than that of the NLBC. The visual acuity of LBC was elevated in 18 eyes (37.5%), 21 eyes (43.75%) without changes, and 9 eyes (18.8%) were decreased. The visual acuity of NLBC was elevated in 29 eyes (65.9%), 9 eyes (20.5%) without changes, and 6 eyes (13.6%) were decreased. A IOFB was removed from the left eye of the patient (Fig. 1.).

**Table 2:** Preoperative visual acuities and visual outcomes of left-behind and guarded group

	No light perception (NLP)	Light perception/ hand move	Count finger~ 0.1 Count finger~ 20/200	0.1~0.4 20/200~20/50	≥0.5 ≥20/40
Preopeartive visual acuities					
LBC group (*n* = 48)	3 (7.5%)	37 (77.1%)	6 (12.5%)	2 (4.2%)	0
NLBC group (*n* = 44)	1 (2.3%)	18 (40.9%)	6 (13.6%)	9 (20.4%)	0
Total	4 (4.3%)	55 (27.5%)	12 (12.5%)	11 (12.0%)	0
Visual outcomes					
LBC group (*n* = 48)	8 (16.7%)	16 (33.3%)	16 (33.3%)	8 (16.7%)	0
NLBC group (*n* = 44)	3 (7.5%)	19 (43.2%)	6 (13.6%)	13 (29.5%)	3 (7.5%)
Total	11 (12.0%)	35 (38.0%)	22 (23.9%)	21 (22.8%)	3 (0.2%)

### Vitreous surgery

**Fig. 2:**
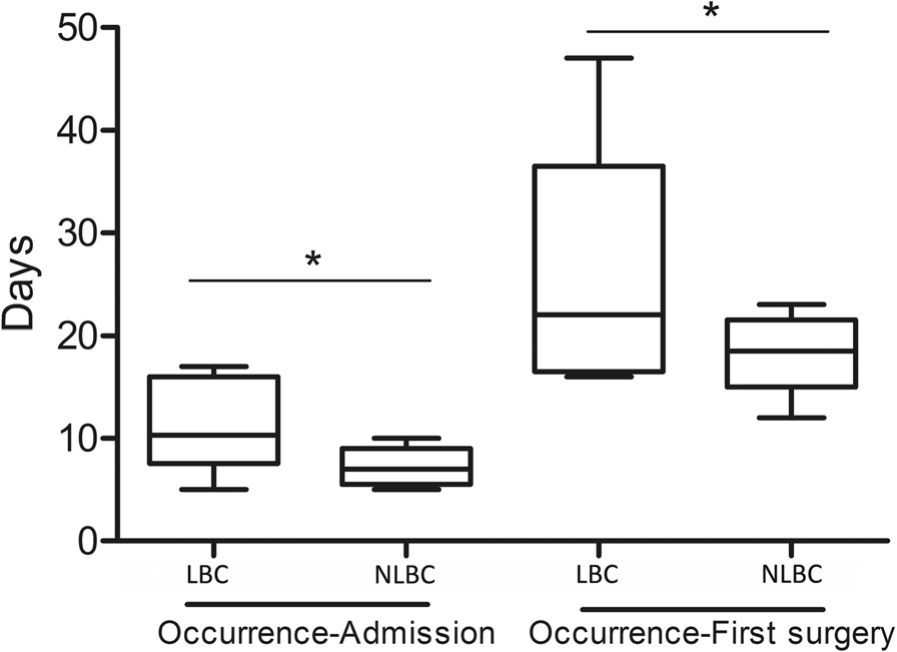
The days from occurrence to admission and from occurrence to first surgery. Data were presented as mean ± SD. The independent t test was conducted to compare the average days from occurrence to admission and from occurrence to first surgery. *p < 0.05 compared with left-behind children

The days from the occurrence of trauma to the hospital admission in LBC group were 9.3 ± 5.7 days, and that in the guarded group were 4.4 ± 2.8 days (Fig. 2.). The length of days from the onset of trauma to the first 25G-PPV in LBC were 35.3 ± 25.1 days, and that in NLBC were 14.3 ± 7.7 days. The length of medical treatment of LBC was significantly longer than that of NLBC.

Vitrectomy was performed on average 2.1 ± 1.0 times, with LBC 2.3 ± 1.0 times and NLBC 1.9 ± 1.0 times. The comparison of vitreous surgery and long-term maintenance status between LBC and NLBC is shown in Table 3. As can be seen from the table, LBC had significantly higher proportion of intraocular lens resection, silicone oil tamponade and endophthalmitis than NLBC. The proportion of LBC who ultimately retain the lens or have the condition to be implanted with IOL was lower than that of NLBC, while the proportion of silicone oil dependence in LBC was higher. In LBC group, there was one case of secondary glaucoma and one case of eyeball enucleation, which were not found in NLBC group.

**Table 3:** Comparison of surgical procedures and postoperative maintenance status

	LBC group (*n* = 48)	NLBC group (*n* = 44)	*P*
Surgical procedures			
Vitrectomy	37 (77.1%)	24 (54.5%)	0.022
Simple pars plana vitrectomy	0	6 (13.6%)	0.010
Lens removal	37 (77.8%)	15 (34.1%)	0.000
Silicone oil tamponade	35 (72.9%)	16 (36.4%)	0.000
C_3_F_8_ (perfluorocarbon) tamponade	3 (6.3%)	13 (29.5%)	0.003
Intraocular antibiotics	18 (37.5%)	3 (6.8%)	0.000
Postoperative maintenance status			
Phakic/pseudophakic eyes	7 (14.6%)	31 (70.5%)	0.000
Silicone oil dependence	24 (50.0%)	9 (20.5%)	0.003
Ahmed valve implantation	3 (6.3%)	1 (2.3%)	0.350
Enucleation of eyeball	3 (6.3%)	0	0.137

Fisher’s exact test was conducted to compare the indexes

## Discussion

In this study, we found that patients in LBC group had more severe ocular posterior segment trauma, lower OTS rating, higher incidence of infective endophthalmitis, more times of surgeries, more complicated surgical procedures, and worse surgical prognosis than those in NLBC.

The LBC often lack complete family guardianship and often live together with their parents, their grandparents, even their parents’ other relatives and friends. Previous study [12] found that the incidence rate of paediatric ocular trauma was higher in rural areas than in urban areas, and the proportion of LBC was much higher than that of NLBC. Children do not have the ability to predict the risk yet, coupled with the ineffective monitoring. Therefore, ocular trauma of these children tends to be heavier and visual function damage is more severe, leading to RAPD ultimately. This study showed that LBC had worse vision before surgery, and the incidence of RAPD (88.9%) was also much higher than NLBC. In addition, the medical and health conditions in rural areas are relatively poor, and the injured eyes often complicated with infection, and the proportion of infectious endophthalmitis is higher. Ocular posterior segment trauma is usually accompanied with lens injury and requires silicone oil tamponade. Excessive injuries and multiple surgical injuries greatly damage the integrity of the eye tissue structure. The results of this study showed that the majority of LBC (77.1%) needed to remove the lens during vitrectomy, and the proportion of silicone oil tamponade (72.9%) was significantly higher than that of the NLBC (34.1%).

Another possible reason why LBC in this study had more severe ocular posterior segment injuries than NLBC was the delayed hospitalization and delayed treatment. The days of LBC’s first admission or first vitreous surgery were significantly more than that of the NLBC. Vitrectomy can reduce the occurrence of retinal detachment and greatly reduce the rate of ocular trauma loss by removing the fibrous scaffold on which proliferative cells depend and removing blood and various growth factors in vitreous body. However, the timing of surgery is very important. The principle of treatment for patients with ruptured eyeball injury is to suture the wound immediately and restore the integrity of the eyeball as soon as possible [13]. In case of suprachoroidal hemorrhage, vitrectomy is considered about 14 days after the injury. At this time, there is a peak of liquefaction in the suprachoroidal hemorrhage, which is easy to drain. The premature operation could not completely drain the accumulated blood, and the delayed operation could result in obvious reaction of mechanical proliferation and increase the difficulty of operation, which was not conducive to the anatomic recovery of posterior segment of the eye. Due to the lack of effective supervision, injuries in LBC are often found late, and the remote location and inconvenient transportation delayed the best treatment time, resulting in higher rate of infectious endophthalmitis, lower OTS rating and worse surgical prognosis.

Mechanical trauma scoring system (OTS) is introduced according to the mechanical trauma registration system (the United States Eye Injury Registry, USEIR) and Hungary mechanical trauma registration system (the Hungarian Eye Injury Registry, HEIR) of multicenter registered mechanical the prognosis of patients with ocular trauma, and on mechanical trauma severity score system [8], now has become a mechanical trauma physician’s guide [14]. In this study, OTS ratings of LBC were poorer, class 1 37.5% of children with serious (18 eyes), far higher than 6.8% of NLBC (3 eyes), as a proportion of class for 3 or more children (10.4%, 5 eyes) with significantly lower than NLBC (29.5%, 13 eyes) (Table 3), which directly led to the worse visual prognosis of LBC. The results suggest that the OTS rating is useful in children to help inform the final prognosis.

In our study, we did not discuss the influence of IOP for two reasons: a) IOP was not available before surgery since open global injury made IOP extremely low, and it was better not to evaluate patients’ IOP in case of pouring of the intraocular structures. b) Postoperative IOPs of all the patients were within the normal range except one patient had high IOP and one patient had atrophy bulbi in the left-behind group. The limitation of this study is the small samples and further study in a large scale is well needed.

## Conclusions

To sum up, this study found that due to the inadequate supervision and delayed medical treatment, left-behind children tend to be seriously injured after ocular trauma and have great risk of infectious endophthalmitis, and generally have poorer surgical prognosis. It is urgent to strengthen the effective care of left-behind children.

## Data Availability

The datasets used and analysed during the current study are available from the corresponding author on reasonable request.

## Abbreviations

LBC: Left-behind children
NLBC: None left-behind children
OTS: Ocular trauma score
PPV: Pars plana vitrectomy
RAPD: Relative afferent pupillary defect
